# Gut-liver axis modulation of *Panax notoginseng* saponins in nonalcoholic fatty liver disease

**DOI:** 10.1007/s12072-021-10138-1

**Published:** 2021-03-03

**Authors:** Yu Xu, Ning Wang, Hor-Yue Tan, Sha Li, Cheng Zhang, Yibin Feng

**Affiliations:** School of Chinese Medicine, Li Ka Shing Faculty of Medicine, The University of Hong Kong, 1/F, 10 Sassoon Road, Pokfulam, Hong Kong S.A.R. People’s Republic of China

**Keywords:** PNS, NAFLD, Steatosis, Fibrosis, Leaky gut, Gut-liver axis, TLR4, Ampkα, Scfas, Gut translocation

## Abstract

**Background and aims:**

Nonalcoholic fatty liver disease (NAFLD) is an obesity-related comorbidity, and it is characterized as a spectrum of liver abnormalities, including inflammation, steatosis, and fibrosis. The gut-liver axis is implicated in the pathogenesis and development of NAFLD. A promising drug agent targeting the gut-liver axis is expected to reverse NAFLD.

**Methods:**

We utilized high-fat diet (HFD)-induced obese mice and obesity-prone *Lep*^*ob*^ mice to examine the gut-liver regulation of the natural medicine *Panax Notoginseng* Saponins (PNS) on NAFLD.

**Results:**

PNS exhibited potent anti-lipogenesis and anti-fibrotic effects in NAFLD mice, that was associated with the TLR4-induced inflammatory signalling pathway in liver. More strikingly, PNS treatment caused a deceleration of gut-to-liver translocation of microbiota-derived short chain fatty acids (SCFAs) products. PNS-induced TLR4 inhibition and restoration of Claudin-1 and ZO-1 proteins in the gut-liver axis contributed to the reverse of leaky gut, which in turn abolished by the addition of lipopolysaccharide (LPS), an agonist of TLR4. Specifically, hepatic steatosis in HFD-treated mice was attenuated by PNS through regulating AMPKα, but restored by the replenishment of LPS. Meanwhile, the anti-fibrotic effect of PNS was abolished by LPS stimulation via the overproduction of collagen I/IV and α-SMA.

**Conclusion:**

PNS exerted hepatoprotection against NAFLD in both ob/ob and HFD-induced obese mice, primarily by mediating the gut-liver axis in a TLR4-dependent manner.

**Graphic abstract:**

**Figure:**
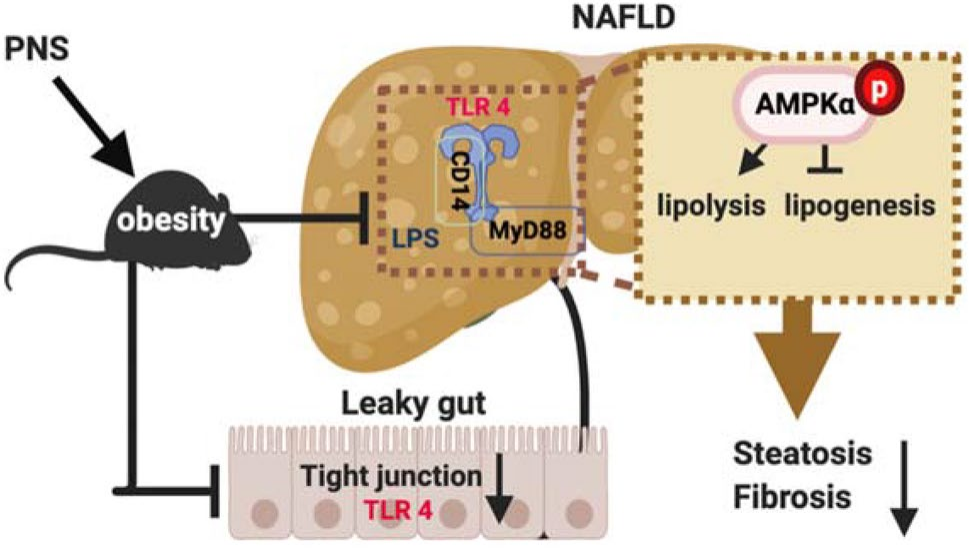
Panax notoginseng saponins (PNS) ameliorated hepatic steatosis and fibrosis, and gut-liver axis-mediated pathogenesis of NAFLD is proposed to occur in a TLR4-dependent manner.

**Supplementary Information:**

The online version contains supplementary material available at 10.1007/s12072-021-10138-1.

## Introduction

Nonalcoholic fatty liver disease (NAFLD) is considered the most common type of chronic hepatic disease, and it occurs in parallel with the global obesity pandemic. During the development and progression of NAFLD, simple hepatic steatosis is considered a benign status, and steatohepatitis (NASH) is the chronic evolution pattern of hepatic steatosis associated with inflammation and fibrosis that can progressively develop into cirrhosis [1]. The multiple-hit pathogenesis of NAFLD has been suggested to be consistently correlated with metabolic derangements in obesity in response to excessive free fatty acid (FFA) buildup and the overproduction of several cellular inflammatory mediators [2].

Recent reports have shown that the interaction of intestinal microbiota, microbial metabolites and gut dysfunction with NAFLD is associated with the inflammatory response [3]. Gut microbiota alterations, gut barrier dysfunction and increased gut permeability promote the translocation of bacterial materials into the liver, which might activate hepatic TLR4 activation. Moreover, TLR4 activation might cause epithelial barrier integrity loss and impair intestinal barrier function via the regulation of intestinal tight junction integrity [4, 5]. During conditions of impaired intestinal barrier function, TLR4 activation mediates gastrointestinal-associated liver disorders. Fatty liver and increased gut permeability may generate a self-perpetuating vicious cycle in which TLR4 activation is involved, namely, gut-liver complex interrelated events in the pathogenesis of NAFLD. Hence, searching for alternative therapeutic approaches to modulate the gut-liver axis via the TLR4 signaling pathway is urgent for implications beyond NAFLD [6].

Recently, research studies pointed out that the natural medicine product *Panax notoginseng* saponins (PNS) exerted potent hepatoprotection against acute ethanol-induced liver injury and CCL_4_-induced hepatic fibrosis [7]. Previously, we proved that PNS has modulating effects on the community structure of gut microbiota [8]. PNS could revert microbiota imbalance and reverse the higher ratio of *Firmicutes/Bacteroidetes* in both diet induced obesity (DIO) mice and ob/ob mice. PNS treatment could increase the abundance of *Parabacteroides distasonis* in ob/ob and DIO mice [8], and *Parabacteroides distasonis* has been proven to be negatively correlated with NAFLD and obesity [9]. However, the underlying mechanism of PNS in obesity-induced NAFLD progression is poorly understood with respect to malfunction of the gut-liver axis. Meanwhile, the effects of PNS on the progression of NAFLD involve the TLR4 signaling pathway, and leaky gut changes need to be confirmed. Therefore, we investigated PNS regulation of hepatic steatosis and fibrosis in *lep*^ob^ mice (ob/ob) and high-fat diet-induced (HFD) obese mice. Specifically, we hypothesized that PNS-modulated gut permeability inhibited the development of hepatic steatosis, inflammation and fibrosis. Here, we provide direct evidence that PNS alleviates NAFLD by decreasing hepatic lipid accumulation and inflammation in response to TLR4 activity and propose a gut-liver axis that mediates the pathogenesis of NAFLD.

## Methods

### Animal experiment

The experimental design and protocols were authorized by the Committee on the Use of Live Animals in Teaching and Research of the University of Hong Kong (CULATR NO. 4357-17). PNS was purchased from China Commercial Company and primarily contain ginsenosides Rb1, Rg1, Re, Rf, Rd and notoginsenoside R1, as shown in Fig. S1. Obese ob/ob mice from The Jackson Laboratory of Bar Harbor received a regular normal diet. Male C57BL/6J mice at 6 weeks old were fed a high-fat diet (60% fat, Research Diets, D12492) throughout the 12-week experimental period. The ob/ob and HFD mice were fed their respective diets for 4 weeks to induce obesity related NAFLD before oral administration of PNS (800 mg/kg per day) or dd water for an additional 8 weeks. The HFD and ob/ob mice were euthanized for the collection of serum, liver and small intestine tissues.

### Cell treatment and transfection

An in-vitro hepatic steatosis model associated with intracellular lipid accumulation and inflammation profiles was established in cultured AML12 hepatocytes induced by palmitic acid. Then, palmitate-induced AML12 hepatocytes were treated with the vehicle or PNS (50 μg/mL) for 24 h. The real-time oxygen consumption rate (OCR) was monitored by a Agilent Seahorse XF^e^ 24 Analyzer, and maximal respiration, proton leakage and basal respiration were calculated to assess PNS-induced dynamic metabolism. AML 12 cells were transfected with mouse siRNA targeting AMPKα 1/2 (sc-45313) or control siRNA (sc-37007) by using Lipofectamine 300 transfection reagent (Invitrogen). As a TLR4 activator, LPS (100 ng/mL) was added to AML 12 cells to detect PNS-induced AMPKα alterations.

### Histology and immunofluorescence

The fixed tissues were dehydrated using a series of ethanol solutions. After cutting into 5-μm sections, the paraffin-embedded slide sections were deparaffinized and rehydrated for hematoxylin and eosin staining (H&E) and Picric Sirius red staining. For immunofluorescence staining, the rehydrated slide sections were blocked with a solution of 5% goat serum and then incubated with α-smooth muscle actin (SMA) antibody (Thermo Fisher, 14-9760-82) overnight at 4 °C in a humidified chamber. After washing, slide sections were incubated with Alexa Fluor-561-conjugated secondary antibody (Invitrogen, USA), and nuclei were stained with 4′-diamidine-2′-phenylindole hydrochloride (DAPI). Immunofluorescence results were visualized and captured via a Carl Zeiss LSM 780 system. For immunohistochemistry, slide sections were blocked with a solution of 5% goat serum and incubated with TLR4 antibody (R&D, MAB1478), followed by incubation with horseradish peroxidase (HRP)-conjugated secondary antibody before visualization by DAB staining. For oil red O staining, the fixed livers were dehydrated in 30% sucrose solution, embedded in OCT and sectioned at 7 μm.

### Identification and quantification of SCFAs by GC–MS/MS analysis

The samples in methanol with 100 mM C2:0-d4 and 50 mM 3-methylvaleric acid internal standard were extracted by bead beating using Precellys homogenization (Bertin Technologies), followed by centrifugation. The supernatant was added with derivatization reagent (DMT-MM and octylamine in methanol) before injection. The identification and quantification of SCFAs were determined through a GC–MS/MS system equipped with SCAN and MRM mode in an Agilent 7890B GC-Agilent 7010 Triple Quadrupole Mass Spectrometer system. The separation of samples was performed through an Agilent DB-5MS capillary column (30 m × 0.25 mm ID, 0.25 μm film thickness) with a flow rate of 1 mL/min. Experimental data were collected by using Process Cleaner and CS launcher software, and the SCFA concentrations were calculated from the standard curves established by different concentrations of chemical standards.

### Quantitative real-time PCR

Total RNA was isolated from the liver tissue and purified by RNA isolated with a total RNA extraction reagent (Vazyme, R401-01), and the RNA quality was assessed by the 260 nm/280 nm measurement ratio. The synthesized cDNA was subjected to PCR, which was performed in triplicate for each sample with ChamQ SYBR color qPCR master mix (Vazyme, Q411-03) on the LC480 platform (Roche, USA). The expression of the targeted gene was normalized to β-actin. Details of the primer sequences are listed in Table S1.

### Western blotting analysis

We extracted proteins from AML12 cells and liver tissues with RIPA lysis buffer, and the protein samples were subjected to centrifugation at 14,000 rpm at 4 °C for 15 min. After quantification, the protein lysates (20 μg) were separated on a 10% SDS–polyacrylamide gel. The separated protein was transferred to a membrane, and the protein membrane was blocked with 5% BSA in TBS-T solution for 2 h. After washing, the blocked membranes were immunoblotted with the targeted antibodies overnight at 4 °C and then incubated with HRP-conjugated secondary antibodies (1:1000). The reactive band signal was visually detected by ECL select substrate (GE Healthcare, Germany) on the Chemidoc chemiluminescent platform (Bio-Rad, USA).

### Statistical analysis

The experimental results are presented as the mean ± standard deviation. Statistical analysis among different groups was carried out by one-way ANOVA or Student’s *t*-test in GraphPad Prism 8.0. Differences among different groups are shown as *p*-values (***p < 0.001, **p < 0.01, *p < 0.05, n.s. not significant).

## Results

### PNS protected against metabolic dysfunction in NAFLD mice

**Fig. 1:**
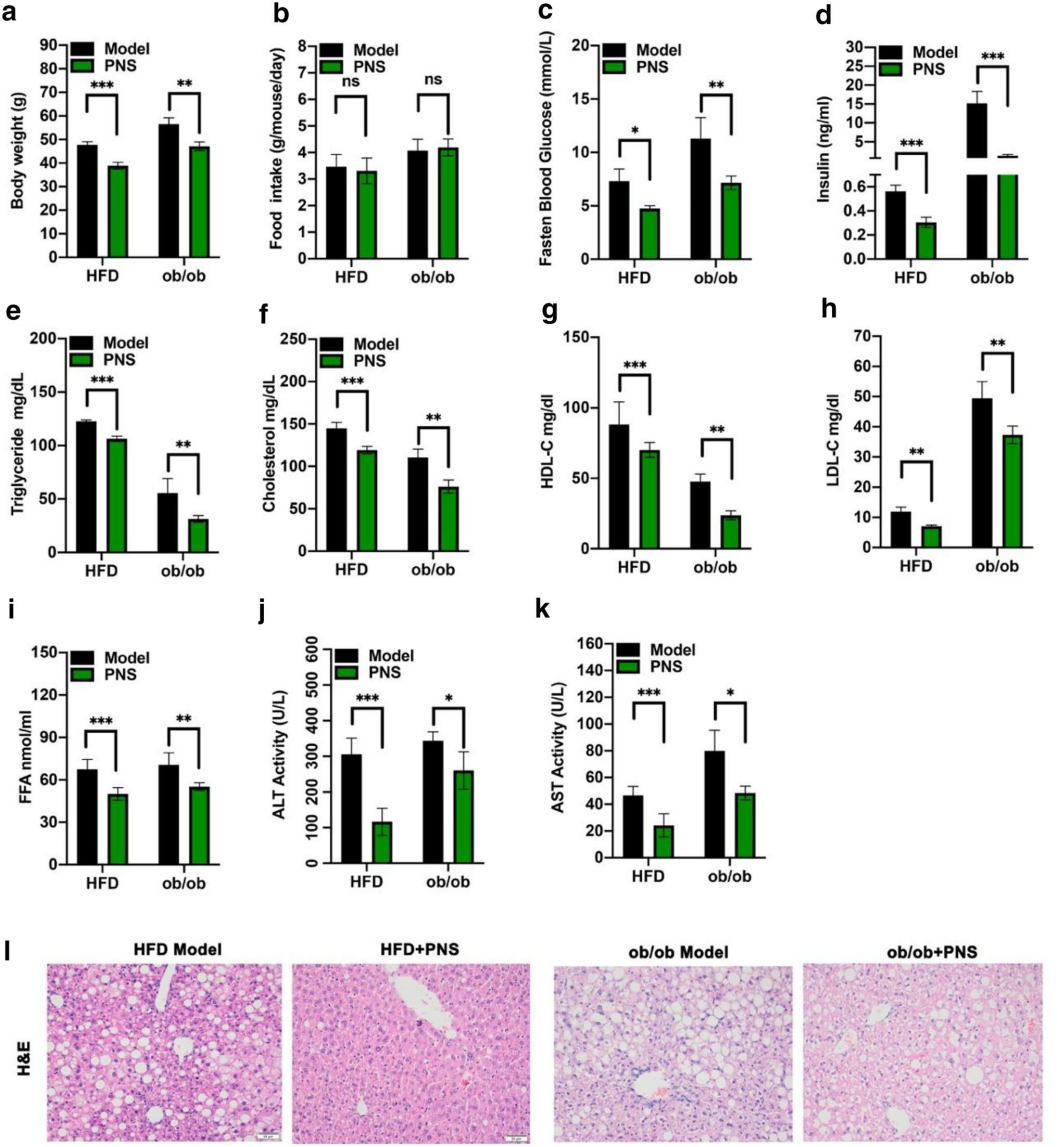
PNS improved metabolic disorders in NAFLD mice. **a** Body weight of HFD and ob/ob mice during vehicle or PNS treatment. **b** Food intake of HFD and ob/ob mice during vehicle or PNS treatment. **c** Fasten blood glucose after 6 h fasting. **d** Serum insulin level of HFD and ob/ob mice during vehicle or PNS treatment. **e**–**i** Serum levels of TG, TC, HLD-C, LDL-C and FFA for the lipid metabolism of HFD and ob/ob mice during vehicle or PNS treatment. **j**–**k** The activities of ALT and AST for the liver function of HFD and ob/ob mice during vehicle or PNS treatment. **l** Representative histological observation by H&E staining of the liver sections (Scale bars, × 50 μm)

As shown in Fig. S2, supplementation with dose-dependent PNS (400 and 800 mg/kg) decreased microvacuolar droplets in the liver sections of ob/ob and DIO mice, indicating that PNS had the capacity to improve the pathological changes of NAFLD. Administration of PNS (800 mg/kg) significantly decreased the body weight of NAFLD mice (Fig. 1a) and showed no significant influence on food intake (Fig. 1b). As shown in Fig. 1c, d, the fasting blood glucose and insulin levels in the serum of NAFLD mice were significantly reduced by PNS treatment. PNS improved glucose metabolic disorders in NAFLD mice. The reduction in TG, TC, HDL-C, LDL-C and FFA levels (Fig. 1e–i) in serum indicated that PNS improved dyslipidemia in NAFLD mice. To investigate the degree of hepatic damage, serum AST and ALT activity (Fig. 1j, k) was measured in NAFLD mice with or without PNS treatment. The results showed that PNS ameliorated AST and ALT activities and reduced hepatic steatosis in NAFLD mice (Fig. 1l).

### PNS alleviated hepatic steatosis in NAFLD mice

**Fig. 2:**
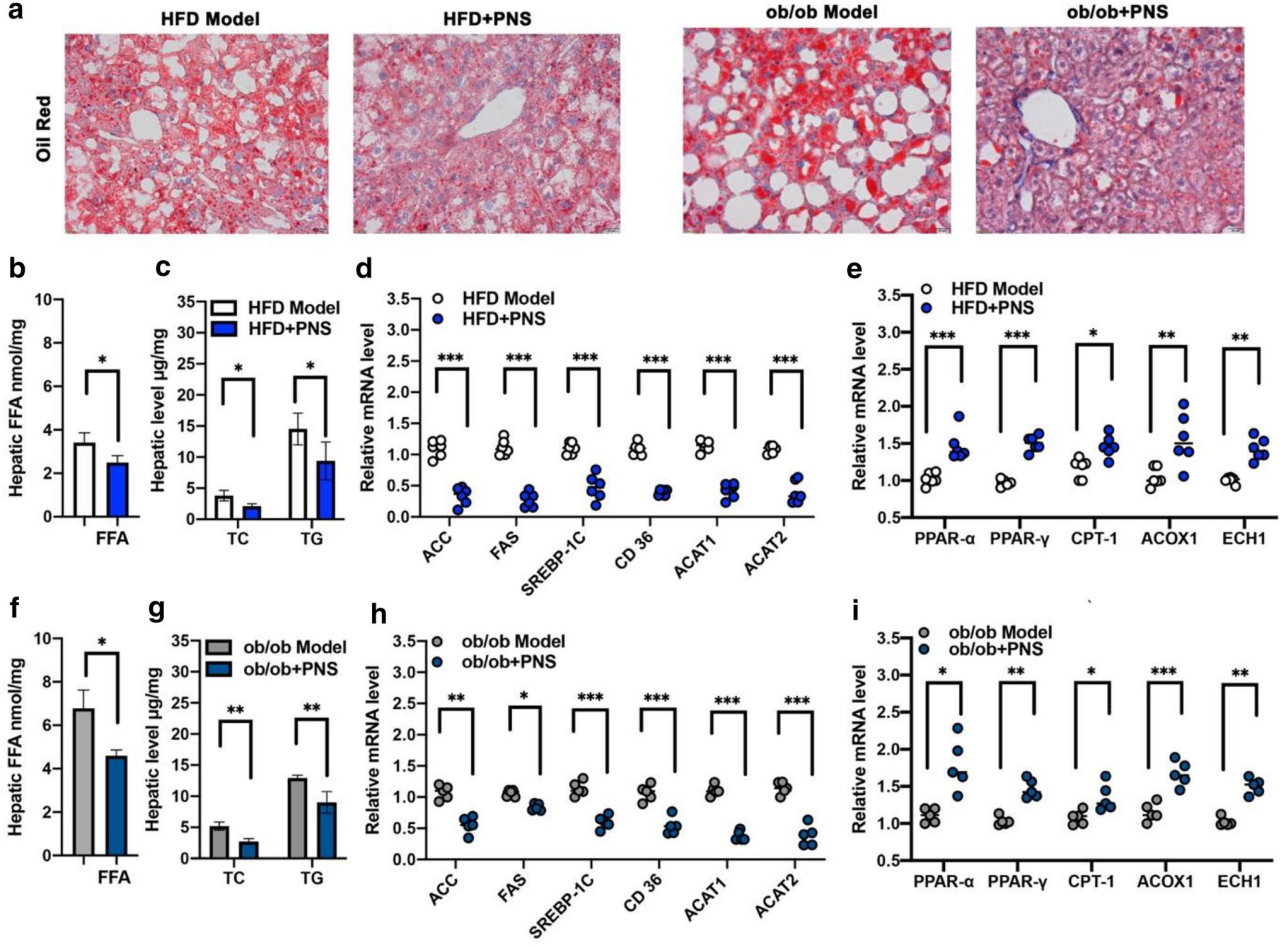
Effects of PNS on hepatic lipogenesis and lipolysis in ob/ob and HFD mice. **a** Representative Oil Red O staining examination in liver sections of HFD and ob/ob mice treated with PNS (800 mg/kg) or vehicle (Scale bars, × 20 μm). **b**, **f** Hepatic FFAs in HFD and ob/ob mice treated with PNS or vehicle. **c**, **g** Hepatic triglyceride and cholesterol in HFD and ob/ob mice treated with PNS or vehicle. **d**, **h** The mRNA expression of lipid metabolism genes, including ACC, FAS, SREBP-1C, CD36, ACAT1 and ACAT2 in HFD and ob/ob mice treated with PNS or vehicle. **e**, **i** The mRNA expression of lipid metabolism genes, such as PPARα, PPARγ, CPT-1, ACOX-1 and ECH1 in HFD and ob/ob mice treated with PNS or vehicle

Oil red O staining showed that PNS treatment reduced the number of hepatic lipid droplets (Fig. 2a) in NAFLD mice. Consistent with the histological examination, decreased levels of hepatic FFAs, TG and TC were observed after PNS treatment in DIO (Fig. 2b, c) and ob/ob mice (Fig. 2f, g). To examine the lipid-lowering mechanism of PNS in hepatocytes, the effects of PNS on fatty acid synthesis, fatty acid transport and fatty acid oxidation were tested in DIO and ob/ob mice, as shown in Fig. 2d, e, h, i. Compared with the model group, the downregulation of lipid accumulation in the liver of the PNS treatment group was involved in multiple aspects of hepatic lipid handling, including lipogenesis (acetyl-CoA carboxylase (ACC), fatty acid synthase (FAS) and sterol regulatory element-binding protein 1 (SREBP-1C)), fatty acid transport (CD36) and fatty acid oxidation (peroxisome proliferator-activated receptor-α/γ (PPARα/γ), carnitine palmitoyltransferase 1 (CPT-1), acyl-CoA oxidase 1 (ACOX-1) and enoyl-CoA hydratase (ECH1)). PNS showed inhibitory effects on fatty acid synthesis and transport by reducing the expression of ACC, FAS, SREBP-1C and CD36 at the mRNA level. Moreover, PNS induced the gene expression of PPARα, PPARγ, CPT-1 and ACOX-1, which promoted fatty acid β-oxidation to inhibit hepatic TG deposition in DIO and ob/ob mice. Additionally, PNS decreased the expression of acyl-CoA cholesterol acyltransferase genes (ACAT1 and ACAT2), which could reduce cholesterol esterification in the liver.

### PNS repressed fibrogenesis in NAFLD mice by inhibiting TLR4 signaling

**Fig. 3:**
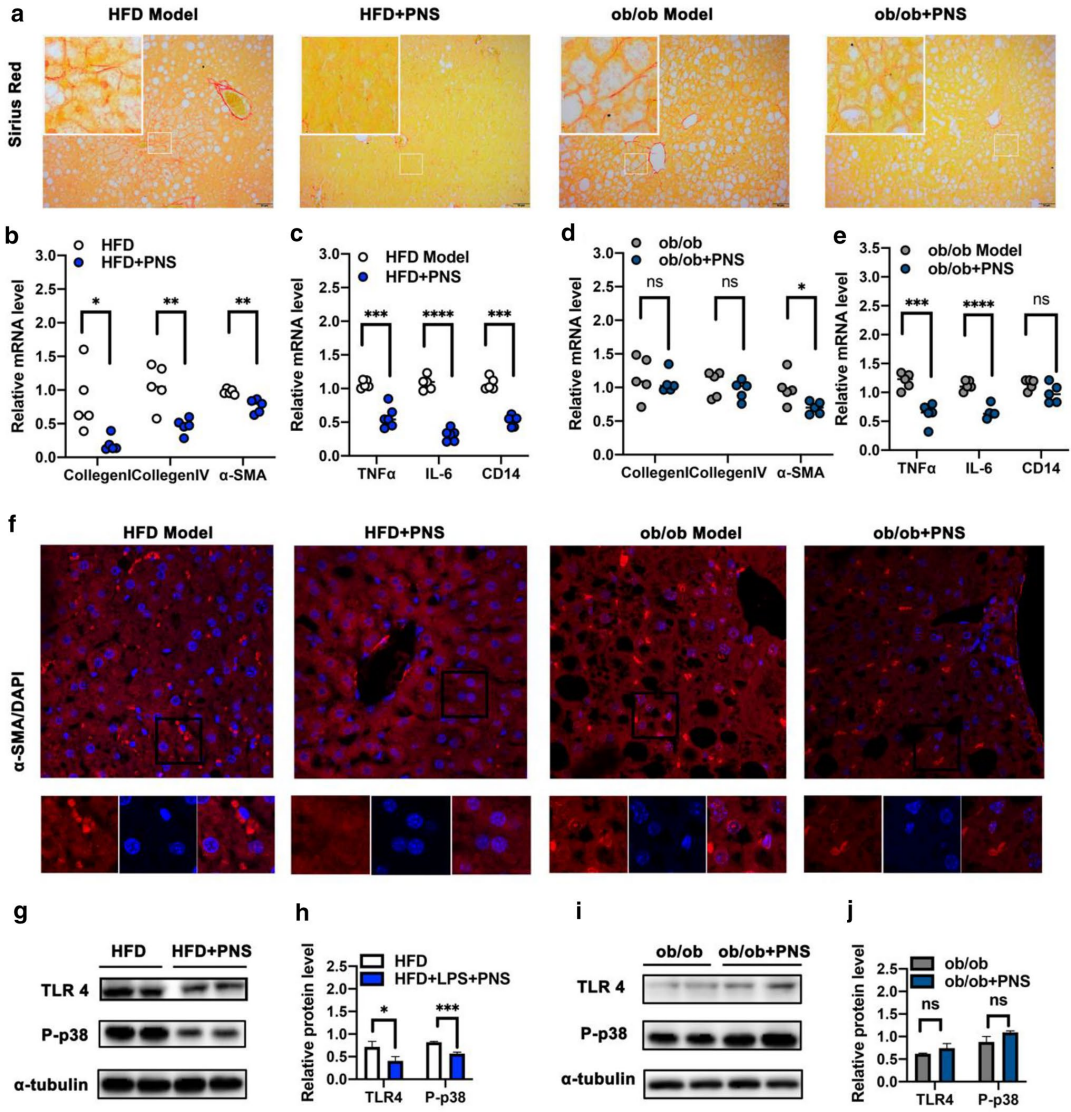
PNS attenuated fibrosis associated with TLR 4 pathway in HFD-fed mice but not ob/ob mice. **a** Representative Sirius Red staining examination in liver sections of ob/ob and HFD mice treated with PNS or vehicle (Scale bars, × 50 μm). **b**, **d** The mRNA expression of fibrosis genes, including Collegen I, Collegen IV and α-SMA in HFD and ob/ob mice treated with PNS or vehicle. **c**, **e** The mRNA levels of inflammatory gens (TNF-α, IL-6 and CD14) in HFD and ob/ob mice treated with PNS or vehicle. **f** Immunofluorescence staining of α-SMA protein (red) staining in HFD and ob/ob mice treated with PNS or vehicle (Scale bars, × 20 μm). **g**–**j** The expression of TLR4 and phosphorylated p-38 proteins in HFD and ob/ob mice were evaluated by the western blotting analysis

As shown in Fig. 3a, the decreased collagen deposition revealed that PNS treatment reduced the extent of hepatic fibrosis in mice. The antifibrotic effect of PNS in DIO mice (Fig. 3b) was associated with the reduced expression of fibrogenesis genes such as collagen I/IV and α-smooth muscle actin (α-SMA). PNS showed a significant influence on the expression of α-SMA in ob/ob mice; however, PNS showed negative effects on the expression of collagen I and collagen IV genes in ob/ob mice (Fig. 3d). α-SMA is a prominent regulator of fibrosis activation, and as shown in Fig. 3f, reduced expression of α-SMA protein was observed after PNS exposure in DIO and ob/ob mice, which agreed with the altered fibrotic gene expression and Sirius red staining results. PNS decreased the expression of tumor necrosis factor α (TNFα) and interleukin-6 (IL-6) at the mRNA level in DIO mice (Fig. 3c) and ob/ob mice (Fig. 3e), and we also assessed the mRNA level of hepatic CD14, which can recognize LPS and regulate responsivity to LPS in the liver [10]. PNS reduced the hepatic CD14 mRNA level in HFD mice (Fig. 3c), decreased the protein expression of TLR4 and phosphorylated p38 (P-p38) (Fig. 3g, h). However, the CD14/TLR4-dependent pathway induced by PNS was not observable in ob/ob mice (Fig. 3e, i, j), suggesting that PNS modulation of CD14/TLR4 activation might involve leptin regulation.

### PNS improved fatty acid-induced dysfunction of lipid metabolism in hepatocytes via TLR4 and AMPKα signaling

**Fig. 4:**
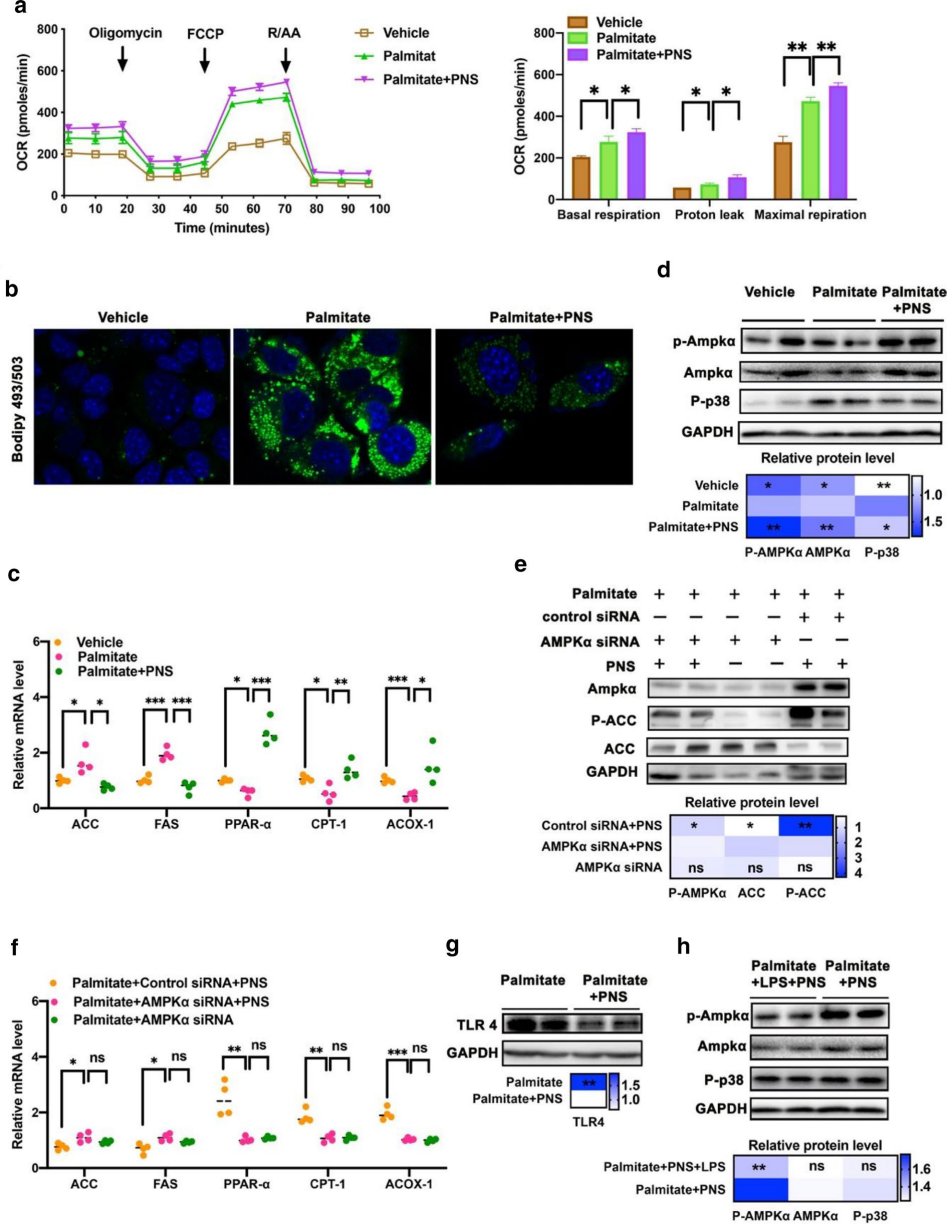
PNS reduced lipid accumulation in palmitate-induced AML12 hepatocytes via TLR4 and AMPKα signalling. **a** Seahorse analysis of oxygen consumption rate (OCR) in AML12 hepatocytes followed by vehicle, palmitate or palmitate plus PNS treatment. **b** Immunofluorescence of lipid droplet (green) in AML12 hepatocytes followed by vehicle, palmitate or palmitate plus PNS treatment. **c** The relative expression levels of ACC, FAS, PPARα, CPT-1 and ACOX-1 mRNA. **d** The protein expressions of AMPKα, phosphorylated AMPKα (P-AMPKα) and p-38(P-p38) in AML12 hepatocytes followed by vehicle, palmitate or palmitate plus PNS treatment. **e** The protein expressions of AMPKα, ACC and phosphorylated ACC (P-ACC) in palmitate-induced AML12 hepatocytes treated with AMPKα siRNA or control siRNA. **f** The relative expression levels of ACC, FAS, PPARα, CPT-1 and ACOX-1 mRNA in palmitate-induced AML12 hepatocytes treated with AMPKα siRNA or control siRNA. **g** The protein expressions of TLR4 in palmitate-induced AML12 hepatocytes treated with PNS or vehicle. **h** The protein expressions of AMPKα, phosphorylated AMPKα (P-AMPKα) and p-38(P-p38) in palmitate-induced AML12 hepatocytes followed by PNS treatment, in combination with LPS or vehicle

As shown in Fig. 4a, the OCR results showed that PNS enhanced both basal and maximal mitochondrial respiration of AML 12 under fatty acid-abundant conditions. AML12 hepatocytes stimulated with palmitic acid (80 μM) showed obvious considerable lipid droplet accumulation, as visualized by BODIPY 493/503 staining (Fig. 4b), whereas culturing palmitic acid-treated AML12 cells in a medium containing PNS supplementation (50 μg/mL) attenuated hepatocellular lipid droplet accumulation. PNS treatment reduced the lipogenesis gene levels of ACC and FAS and increased the lipolysis gene levels of PPARα, CPT-1 and ACOX-1 in hepatocytes (Fig. 4c). PNS supplementation enhanced the phosphorylation of AMP-activated protein kinase α (P-AMPKα) (Fig. 4d). Moreover, we found that AMPKα knockdown blocked the phosphorylation of ACC in PNS-treated cells (Fig. 4e) and counteracted the effects of PNS on the expression levels of the ACC, FAS, PPARα, CPT-1 and ACOX-1 genes (Fig. 4f). The inhibition of TLR4 and phosphorylated p38 also contributed to the PNS-mediated reduction in lipid deposition (Fig. 4d, g) in palmitate acid-stimulated AML12 hepatocytes. To address whether PNS-mediated TLR4 activity is associated with AMPKα activation, we used LPS (100 ng/ml) as a TLR4 activator in PNS-treated hepatocytes, and LPS exposure decreased the PNS-induced AMPKα phosphorylation (Fig. 4h). These results indicated that the inhibitory effects of PNS on TLR4 signaling were beneficial for AMPKα signaling activation.

### PNS altered gut lipid metabolite profiles and gut translocation of SCFAs to the liver

**Fig. 5:**
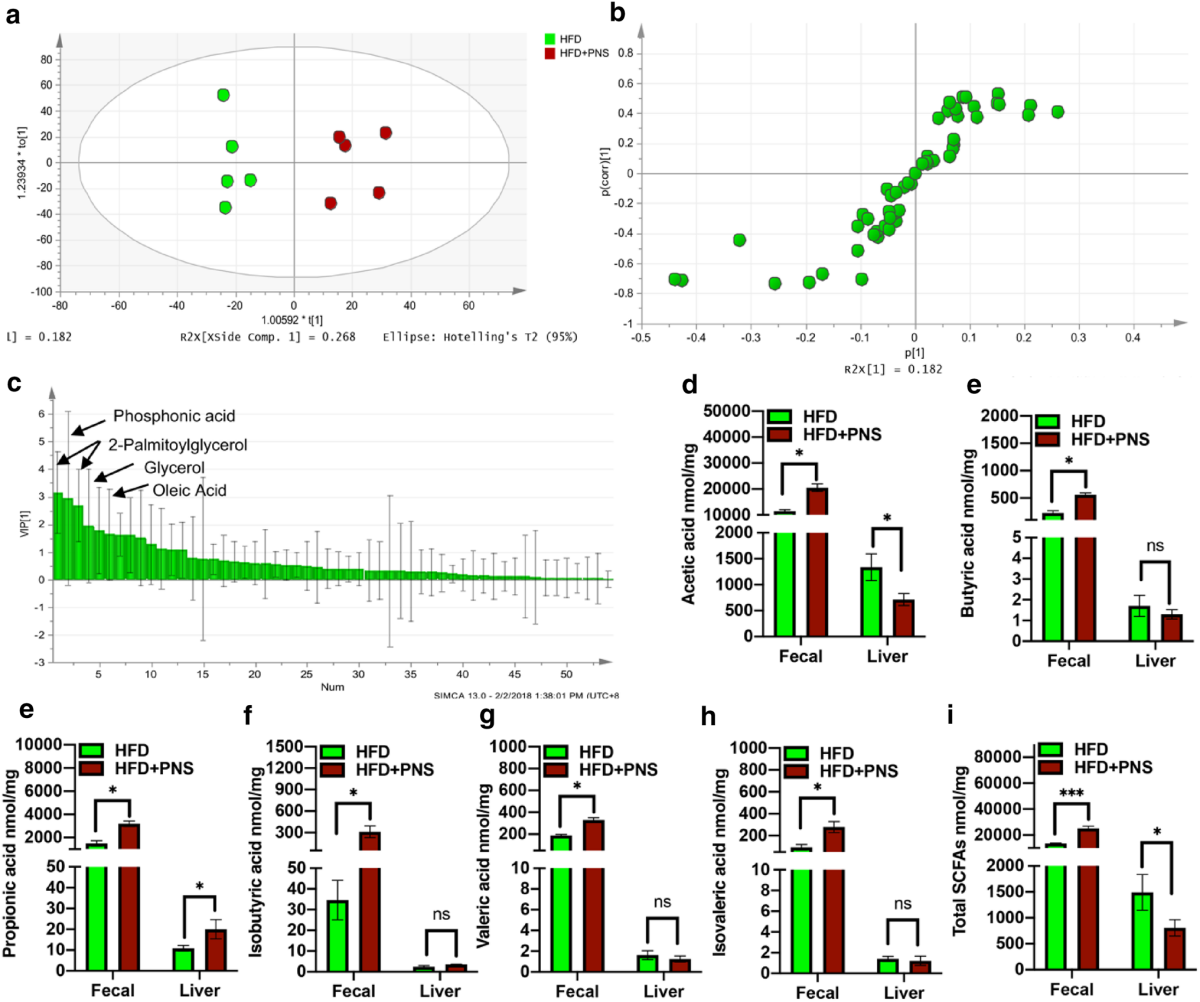
PNS influenced fatty acid metabolic profiles in gut-liver axis based on GC–MS/MS analysis. **a** Orthogonal partial least-squares discriminant analysis (OPLS-DA) score plot showing group separation in fecal metabolic profile of HFD and HFD + PNS groups. **b** The OPLS-DA loading S-plot of the HFD and HFD + PNS groups. **c** Lipid metabolites with VIP > 1.0 in the HFD and HFD + PNS groups are shown with names. **d**–i Major short-chain fatty acid levels (based on dry weights) were quantified in fecal materials and liver from the HFD mice exposed to vehicle and PNS

Based on the resulting GC/MS analysis data, multivariate analysis by SIMCA-P + software 12.0 was performed to elucidate the patterns of gut metabolic profiles. Orthogonal partial least-squares discriminant analysis (OPLS-DA) and the loading of S-plot data were performed to compare metabolic variants between the HFD-fed model and HFD + PNS groups. The results of OPLS-DA analysis showed a different clustering of lipid metabolite features in the stool of DIO mice after PNS treatment (Fig. 5a). The loading S-plot showed a clear separation of distinct altered lipid materials between the HFD Model and the HFD + PNS groups (Fig. 5b). GC/MS analysis identified significant alterations in dietary lipid materials, including glycerol, oleic acid and 2-palmitoyglycerol, after PNS exposure in HFD-fed mice (Fig. 5c). These results indicated that PNS significantly changed the dietary lipid metabolite profile in the gut of DIO mice.

Specifically, in the fecal samples of PNS-treated HFD mice (HFD + PNS), there was a significant increasing trend in the amount of SCFAs, including acetic acid, butyric acid and propionic acid (Fig. 5d–i). We next determined whether PNS influences SCFA absorption into the liver. Nevertheless, compared with the HFD-fed model, PNS-treated mice had a significant decrease in the hepatic level of acetic acid and an increase in the level of propionic acid, but there was no substantial change in the amount of butyric acid, isobutyric acid, valeric acid, and isovaleric acid after PNS treatment (Fig. 5d–i). Comparing the total amount of SCFAs between the HFD Model and HFD + PNS groups, PNS treatment increased the total amount of SCFAs in the gut but decreased the total content in the liver. PNS treatment altered gut lipid metabolism and prevented gut translocation of SCFAs into the liver, subsequently protecting the liver from lipid overload.

### PNS improved gut leakage by repressing TLR4 activation in the intestines

**Fig. 6:**
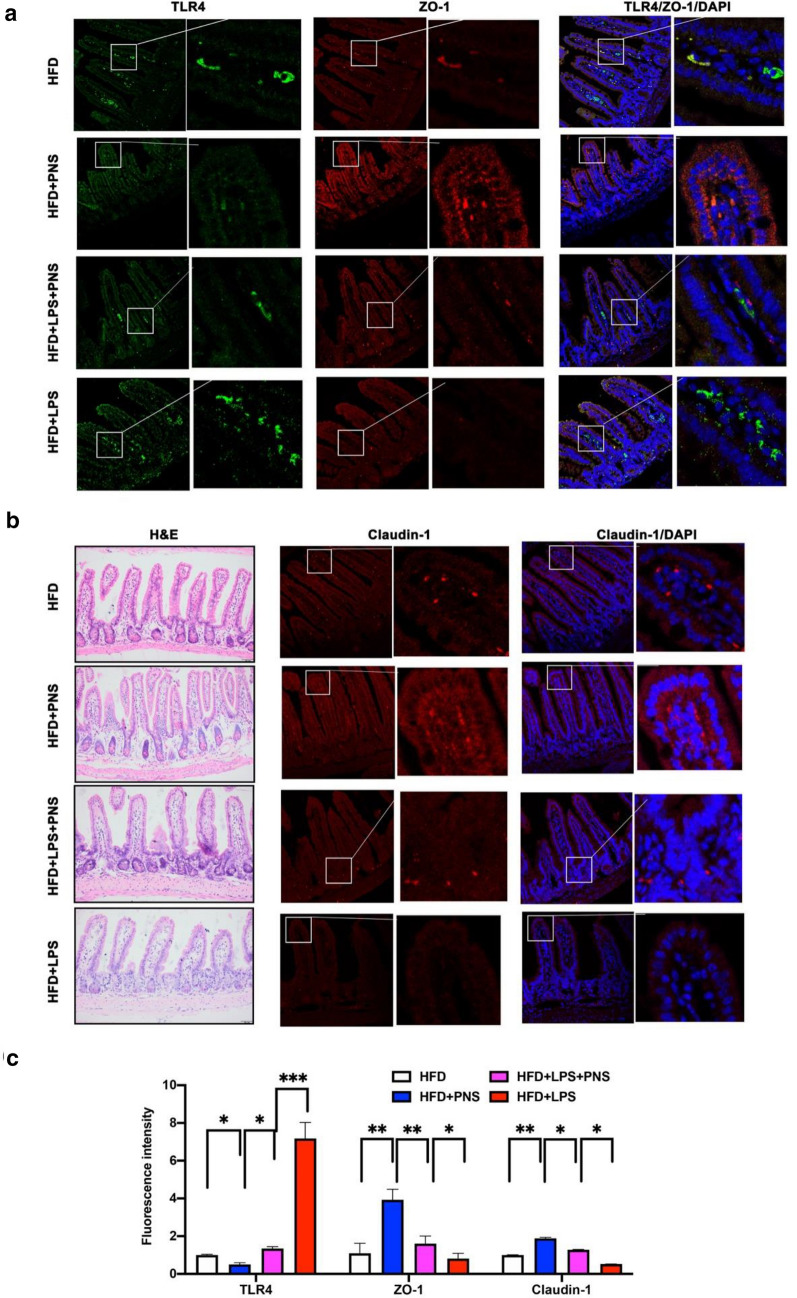
PNS decreased leaky gut via increasing tight junction proteins and decreasing TLR 4 activity. **a** Immunofluorescence staining of TLR4 and ZO-1staining in small intestine of HFD, HFD + PNS, HFD + LPS and HFD + LPS + PNS groups (Scale bars, × 50 μm). **b** Histopathological observation of small intestine by H&E staining (Scale bars, × 50 μm) and immunofluorescence of Claudin-1staining in small intestine of each group (Scale bars, × 50 μm). **c** Quantified fluorescence intensities in **a** and **b**

To address whether PNS interferes with TLR4 activation in the gut, we measured TLR4 expression in the small intestine after PNS treatment. PNS significantly inhibited TLR4 expression in the small intestine and improved intestinal permeability by increasing the expression of tight junction proteins (zonula occludens 1 (ZO-1) and claudin-1) (Fig. 6a, b). To clarify whether PNS-modulated intestinal permeability is dependent on TLR4 activation, we used LPS as an activator of TLR4 in the progression of gut leakage. The LPS-induced increase in gut permeability has been proven to be mediated by TLR-4–dependent activation [11]. After feeding a HFD for 4 weeks, the HFD-fed mice were simultaneously treated with PNS for an additional 8 weeks in combination with LPS supplementation (i.p., 0.25 mg/kg). After LPS supplementation, TLR 4 expression and gut permeability were increased in HFD + LPS mice, as shown in Fig. 6. The improved gut barrier observed in PNS-treated mice was correlated with the inhibition of TLR 4 in both HFD and HFD + LPS mice. Compared with PNS-treated HFD-fed mice (HFD + PNS), HFD + LPS + PNS mice showed degenerated villus structures, damaged crypts, decreased amounts of goblet cells and neutrophil infiltration in ileal sections of the small intestine. LPS exposure reactivated TLR4 expression, which led to less improvement in gut permeability in HFD + LPS + PNS mice. LPS supplementation reversed PNS-mediated TLR4 deactivation and blocked the improvement of gut permeability, which was further confirmed by decreasing the expression of ZO-1 and claudin-1 proteins in HFD + LPS + PNS mice (Fig. 6a, b).

### TLR4 reactivation attenuated the anti-NAFLD effect of PNS

**Fig. 7:**
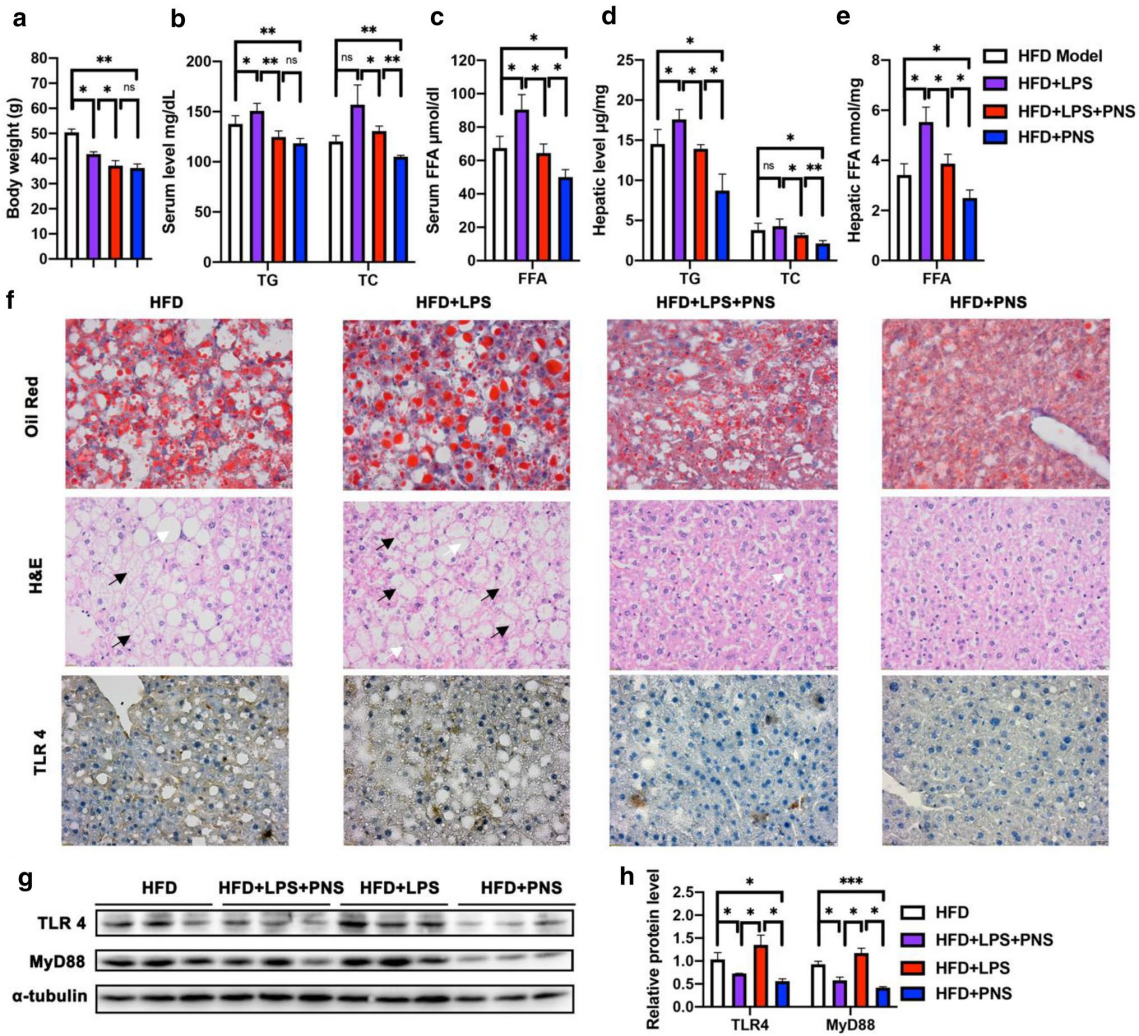
LPS exposure made interference with the protective effects of PNS on NAFLD. **a** Body weight assessment of HFD, HFD + PNS, HFD + LPS and HFD + LPS + PNS groups. **b**, **c** Serum TG, TC and FFA of each groups. **d, e** Hepatic TG, TC and FFA levels. **f** Representative Oil Red and H&E staining examination. Black arrow heads show infiltration of inflammatory cells and white arrowheads indicate lipid droplet, and immunohistochemical staining of TLR-4 in liver sections in each groups (Scale bars, × 20 μm). **g**, **h** The relative protein expression of TLR4 and MyD88 in HFD, HFD + PNS, HFD + LPS and HFD + LPS + PNS groups

As shown in Fig. 7a–e, LPS supplementation in HFD + LPS mice led to a significant alteration in body weight and exacerbated lipid metabolic disorders in the serum and liver. The LPS-induced weight reduction might contribute to its action on vagal afferent neurons, resulting in hypophagia. However, LPS exposure in PNS-treated HFD mice showed no significant influence on the changes in body weight (Fig. 7a) or serum TG levels (Fig. 7b). Nevertheless, the LPS exposure in PNS-treated HFD mice reversed PNS-mediated inhibition of the production of TC and FFA in serum (Fig. 7b). Moreover, LPS addition increased the levels of hepatic TG, TC and FFAs in PNS-treated mice (Fig. 7d, e), which indicated that LPS exposure obstructed the effects of PNS on the progression of NAFLD. Subsequently, the observed results from H & E and oil red O staining (Fig. 7f) showed that the increased lobular inflammation, hepatocyte ballooning and large fat droplets induced by HFD plus LPS were decreased by PNS administration. However, compared with the HFD + PNS group, the HFD + LPS + PNS group had increased hepatic lipid accumulation and hepatic TLR4 protein expression, as shown by immunochemical staining (Fig. 7f). The increased expression of TLR4 and MyD88 protein detected by immunoblotting (Fig. 7g) further confirmed that PNS treatment of NAFLD was associated with the LPS-induced TLR4/MyD88 signaling pathway.

**Fig. 8:**
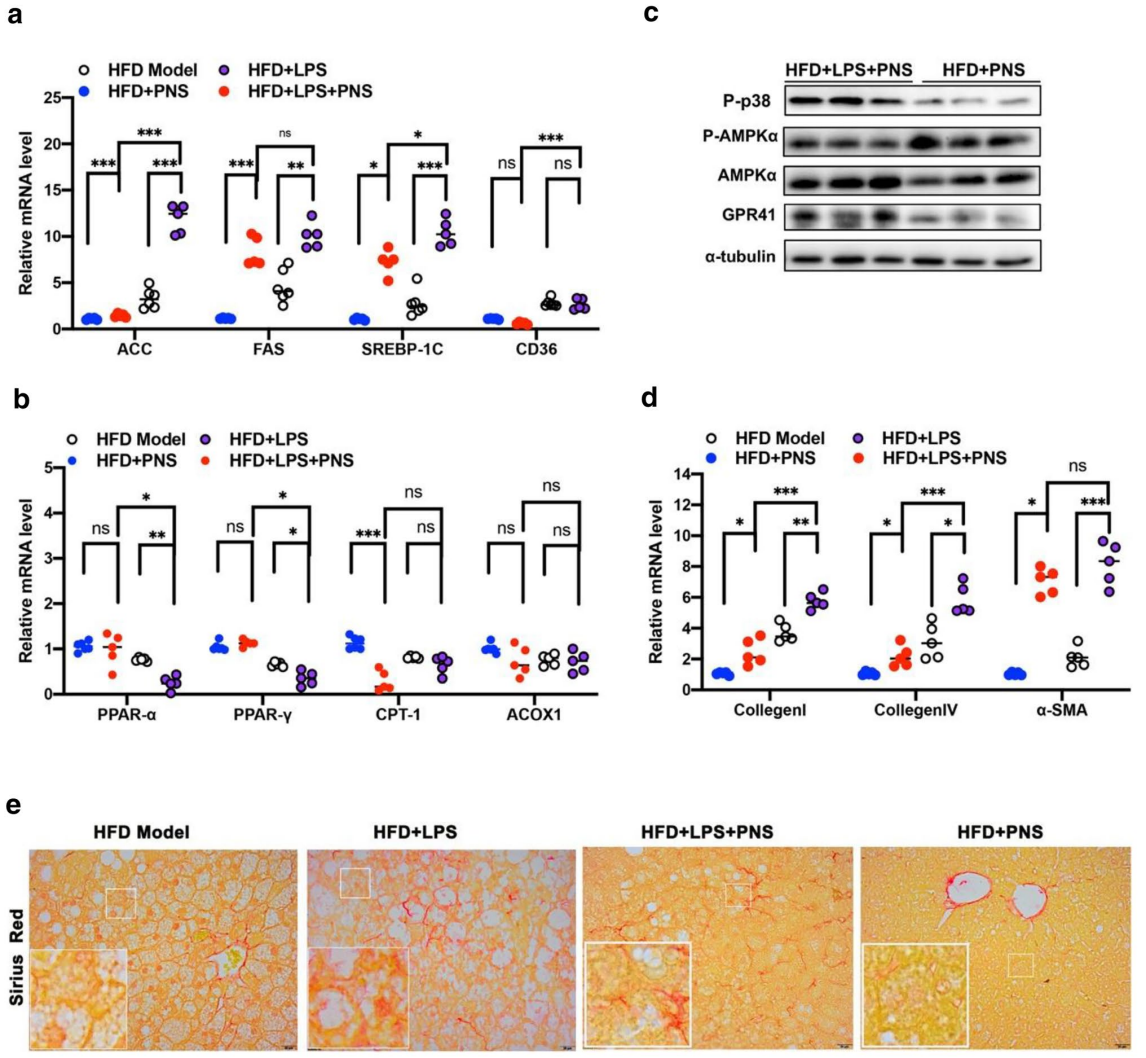
LPS exposure made interference with the protective effects of PNS on NAFLD. **a** The relative mRNA levels of ACC, FAS, SREBP-1C and CD 36 genes expression in HFD, HFD + PNS, HFD + LPS and HFD + LPS + PNS groups. **b** The relative mRNA levels of PPARα, PPARγ,CPT-1 and ACOX-1 expression in HFD, HFD + PNS, HFD + LPS and HFD + LPS + PNS groups. **c** The protein expression of TLR4, phosphorylated p38, phosphorylated AMPKα, AMPKα and GPR41 in HFD + PNS and HFD + PNS + LPS groups. **d** The relative mRNA expression of CollagenI, CollagenIV and α-SMA in HFD, HFD + PNS, HFD + LPS and HFD + LPS + PNS groups. **e** Representative Sirius Red staining of liver sections in HFD, HFD + PNS, HFD + LPS and HFD + LPS + PNS groups. (Scale bars, × 20 μm)

As shown in Fig. 8a, b, LPS exposure modulated fatty acid synthesis and consumption by changing the levels of ACC, FAS, SREBP-1C and PPARα/γ in NAFLD mice. LPS invasion significantly abolished the effects of PNS on steatosis by increasing the expression of lipogenesis genes, including ACC, FAS, and SREBP-1C (Fig. 8a), and decreased the expression of lipolysis genes such as CPT-1 (Fig. 8b). LPS supplementation in HFD + PNS mice increased the protein expression of phosphorylated p-p38 and GPR41 and decreased the protein expression of phosphorylated AMPα in liver tissues (Fig. 8c). Moreover, LPS exposure promoted the mRNA levels of collagen I/V and α-SMA, and LPS reversed the PNS-induced reduction in the expression of these fibrogenesis genes in the HFD + LPS + PNS group (Fig. 8d), which means that LPS restored the antifibrotic effects of PNS, and this influence was observed visually by Sirius red staining (Fig. 8e).

**Fig. 9:**
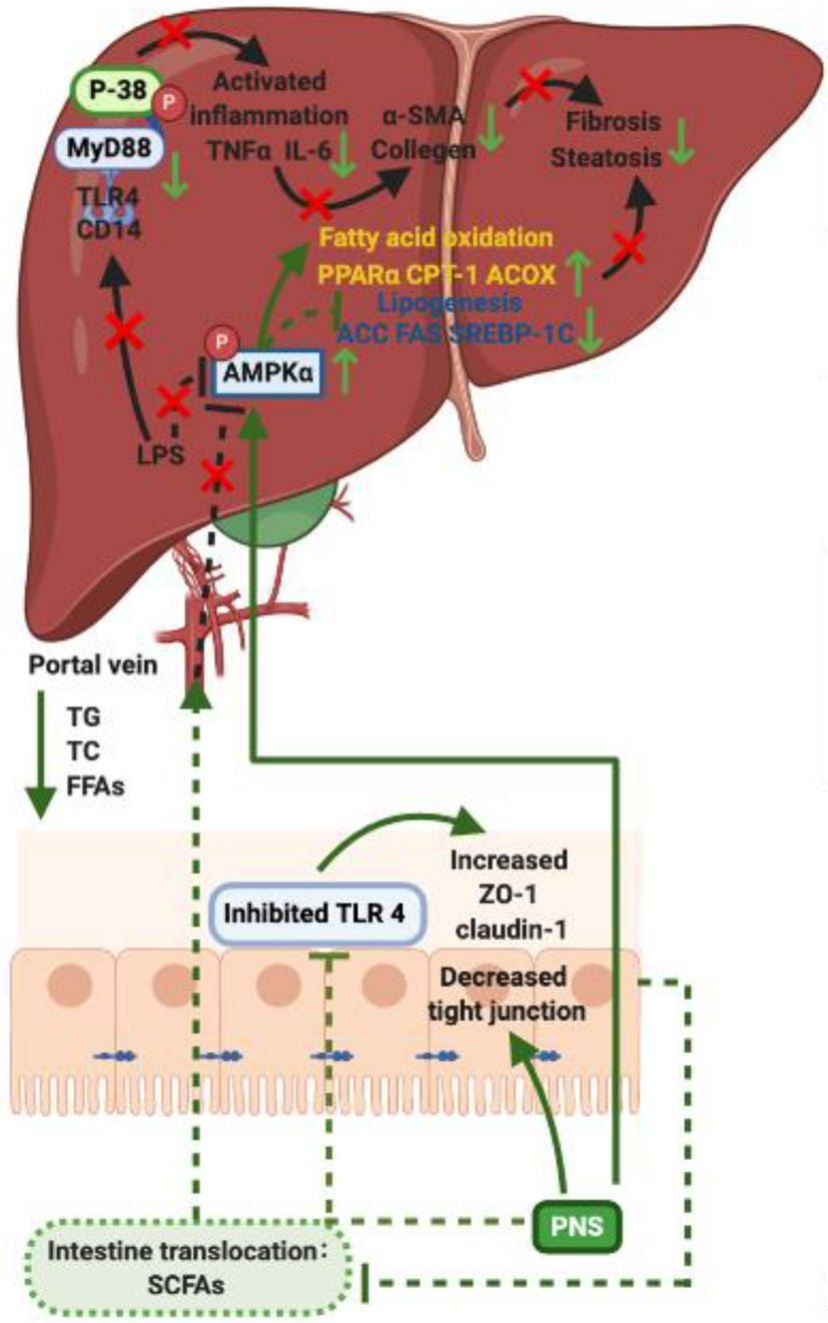
PNS modulated gut-liver axis in NAFLD via interference with LPS-induced TLR 4 and AMPKα signalling pathway. PNS improved the gut intestinal barrier at multi-pathways including enhancement of intestinal tight junction permeability and inhibition of TLR-4 inflammatory response. These resulted in decreased translocation of microbial production (SCFAs) from gut to liver through the portal circulation. In addition to decreased levels of FFAs, the AMPKα signalling pathway was activated, and fatty acid oxidation was promoted by PPARα, CPT-1 and ACOX, as well as lipogenesis was reduced via ACC, FASS and SREBP-1C. PNS blocked LPS induced-CD14-TLR-4 activation in liver where inhibited the production of TNFα and IL-6, further improved hepatic fibrosis via α-SMA and Collagen I/IV

## Discussion

NAFLD is a benign disease associated with obesity, hyperglycemia, dyslipidemia, and insulin resistance. PNS has been described to possess anti-insulin resistance properties, ameliorate glucose intolerance and reduce obesity-induced oxidative stress [12] in obese mice. At present, the clinical trials recorded in the U.S. National Library of Medicine have mentioned the clinical application of *Panax notoginseng* in treating hyperlipidemia (NCT04069715) and obesity (NCT03654391), providing potential human evidence that supported our findings regarding use of PNS in obesity-related NAFLD. In the current study, PNS showed beneficial effects on NAFLD, as evidenced by the diminution of hepatic triglyceride accumulation and hepatic fibrogenesis in DIO and ob/ob mice. In this regard, our findings indicated that PNS influenced the main processes of fatty acid production and oxidation, which determined the flux of hepatic lipids. PNS could modulate the production of fatty acids via de novo fatty acid synthesis genes (ACC, FAS and SREBP-1C) and break them down via fatty acid oxidation genes (PPARα, CPT-1 and ACOX-1). The high level of lipid oxidation induced by PNS was also supported by the enhanced oxygen consumption rate (OCR) in PNS-treated hepatocytes under fatty acid-abundant conditions (Fig. 9).

Our mechanistic studies in HFD-induced NAFLD mice potentially implied that PNS supplementation improved hepatic steatosis and fibrosis via inhibition of hepatic CD14 and TLR4 activation. Under pathological conditions, TLR4 can induce proinflammatory signaling such as the p38 pathway [13, 14], which induces the production of inflammatory cytokines (e.g., IL-6 and TNF-α) from Kupffer cells, ultimately leading to profibrogenic signals. Our studies showed the inhibitory effects of PNS on TLR4-mediated inflammation, which improved hepatic fibrosis in DIO mice. Furthermore, the effects of PNS on hepatic CD14 and TLR4 expression in ob/ob mice were also investigated. Despite exhibiting improvement of steatosis, PNS produced no significant change in hepatic CD14 and TLR4 expression in ob/ob mice. Furthermore, compared with the antifibrotic effects of PNS on DIO mice, PNS had less effective action on hepatic fibrosis in ob/ob mice, which might be due to the negative influence of PNS on CD14 and TLR4 activation. Previous studies have revealed that LPS-CD14-induced TLR4 activation in liver inflammation and fibrosis occurs in a leptin-dependent manner. Leptin deficiency can induce hepatic CD14 reduction in ob/ob mice, resulting in a deterioration of the Kupffer cell blockade. These findings might explain why PNS repressed fibrogenesis via inhibition of TLR4 signaling in HFD-fed mice but not ob/ob mice. Moreover, the CD14/TLR4-dependent pathway induced by PNS was not observable in ob/ob mice, demonstrating that PNS interference with TLR4/CD14 activation may be associated with leptin regulation.

Additionally, the mucosal TLR4-induced MYD88 signaling pathway contributes to the development of hepatic steatosis. To manage effective treatment options for gastrointestinal-associated liver diseases by developing new drugs, we elucidated the cross-talk between TLR4-induced inflammation and potential therapeutic medicines that facilitate the interaction between the gut and the liver. Our study verified the metabolic benefits of PNS and provided clues about the PNS-induced cross-talk between the gut and liver for protection against NAFLD.

However, whether PNS interference is due to a direct effect or is secondary to gut leakiness in decreasing hepatic steatosis and fibrosis warrants further investigation. We confirmed that PNS supplementation reduced lipid lipogenesis in palmitate-induced hepatocytes and that LPS could block PNS interference on lipid accumulation in hepatocytes. To address how the inhibition of TLR4 contributed to the reduced lipid deposition, AMPKα, as an essential cellular energy sensor in lipid metabolism, was detected. Our results showed that PNS-induced AMPKα activation reduced lipogenesis in hepatocytes and that this promotion could be interrupted by TLR4 activation, which means that the inhibitory effects of PNS on TLR4 have beneficial effects on AMPKα regulation, resulting in the improvement of hepatic lipid metabolism. Next, our research aimed to confirm whether PNS modulation of the TLR4 signaling pathway involved gut-liver axis malfunction. Accumulating evidence in both animals and humans has indicated that increased intestinal permeability (leaky gut) facilitates the translocation of microbial products, including SCFAs, across tight junctions into the liver [15], which could trigger the progression of NAFLD [16, 17]. Our studies confirmed that the total contents of FFAs and TG were decreased after PNS treatment, which suggests that PNS can induce FFA changes to improve NAFLD. The significant differences in the gut levels of LCFAs (oleic acid, 2-palmitoyglycerol) and SCFAs (acetic acid, butyric acid, propionic acid, etc.) after PNS exposure in HFD-fed mice suggested that PNS might influence the changes in the fatty acid composition and content in the gut of mice. A notable finding of this study was the PNS-induced decreasing trend in hepatic SCFAs, as well as increased SCFA production in the small intestine observed in DIO-induced NAFLD mice, which indicated that an indirect effect of PNS on NAFLD progression was associated with gut permeability improvement by increasing the expression of tight junction-associated proteins (ZO-1 and Claudin-1). The reason that PNS increased the levels of SCFAs in the gut might be associated with the regulation of PNS on increasing the level of SCFA-producing bacteria. Due to the improvement of leaky gut induced by PNS, the large amount of SCFAs has difficulty entering the liver to activate G-protein coupling receptors such as GPR41, further mediating steatosis and inflammation [18]. In the current studies, our results from HFD mice underscored a contributing role of TLR4 activation in elevating gut leakiness [4, 19], given that PNS decreased TLR4 expression and activated tight junction proteins (ZO-1 and claudin-1). We observed a significant up-regulation of TLR4 expression in the intestine of LPS-treated mice in the presence of PNS, suggesting that LPS can reverse the inhibitory effect of PNS on TLR4 expression in the intestine. Indeed, LPS treatment can activate TLR4, which was responsible for the increase of gut permeability [20]*.* LPS treatment abolished the improvement of gut permeability by PNS. Although the recovery of TLR4 expression in PNS-treated mice using LPS as an antagonist of PNS can completely conclude that the effect of PNS is solely dependent on TLR4 activation, as LPS may have some off-target effect, the findings of our study combined with literature report may somehow indicate that the improvement of gut permeability induced by PNS is, partially if not all, associated with PNS mediated-TLR4 deactivation. Indeed, PNS modulation was relevant in the activation of TLR4 elicited by LPS exposure in both liver and intestinal tissues of HFD mice, as supported by the results that LPS weakened the modulatory effects of PNS on NAFLD. LPS exposure in HFD mice with PNS administration led to exacerbated steatosis by decreasing AMPKα activation and increasing collagen I, collagen IV and α-SMA, which reversed the antifibrotic activation of PNS. The interference of LPS on the anti-NAFLD effect of PNS confirmed that the improvement of NAFLD induced by PNS was associated with the TLR4 pathway.

## Supplementary Information

Below is the link to the electronic supplementary material.

Supplementary file1 (DOCX 12139 KB)

## Abbreviations

NAFLD: Nonalcoholic fatty liver disease
PNS: *Panax notoginseng* Saponins
LPS: Endotoxemia
TLR4: Toll-like receptor
MyD88: Myeloid differentiation primary response 88
TG: Triglyceride
TC: Cholesterol
FFA: Free fatty acid
AST: Aspartate aminotransferase
ALT: Alanine aminotransferase
ACC: Acetyl-CoA carboxylase
PPARγ: Peroxisome proliferator-activated receptor-alpha
SREBP-1C: Sterol regulatory element-binding protein 1
OPLS-DA: Orthogonal partial least-squares discriminant analysis
SCFAs: Short fatty acids
HFD: High-fat diet
α-SMA: α-Smooth muscle actin
TNFα: Tumor necrosis factor α
IL-6: Interleukin-6
CPT-1: Carnitine palmitoyltransferase I
ACOX-1: Acyl-CoA oxidase 1
AMPKα: AMP-activated protein kinase α
ZO-1: Zonula occludens 1
