# Supplementary material for: Gut-liver axis modulation of Panax notoginseng saponins in nonalcoholic fatty liver disease
Source: Hepatol Int. 2021 Mar 3;15(2):350–65. doi: 10.1007/s12072-021-10138-1 (PMC8144126; doi:10.1007/s12072-021-10138-1)
Supplement: Supplementary file 1 — Supplementary file1 (DOCX 12139 KB) [file 12072_2021_10138_MOESM1_ESM.docx]

**
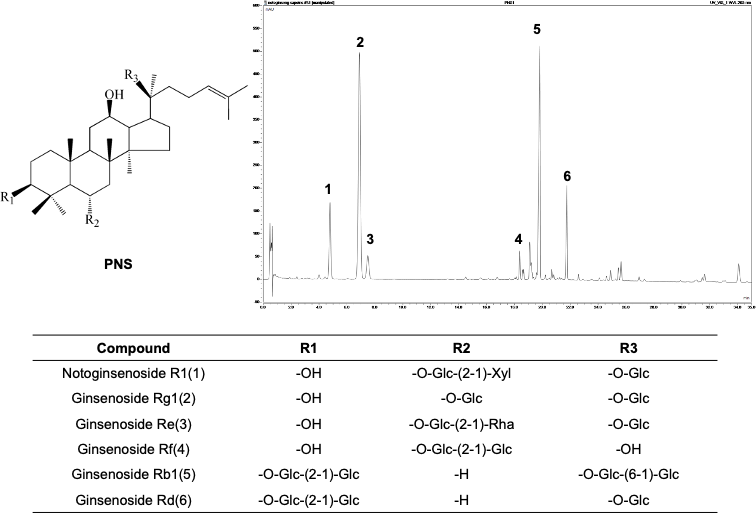
**

**Figure S1** **UPLC-UV profiles Chemical structure of compounds in PNS**. (1) Notoginsenoside R1(2) Ginsenoside Rg1(3) Ginsenoside Re (4) Ginsenoside Rf (5) Ginsenoside Rb1 (6) and Ginsenoside Rd contained in PNS sample.


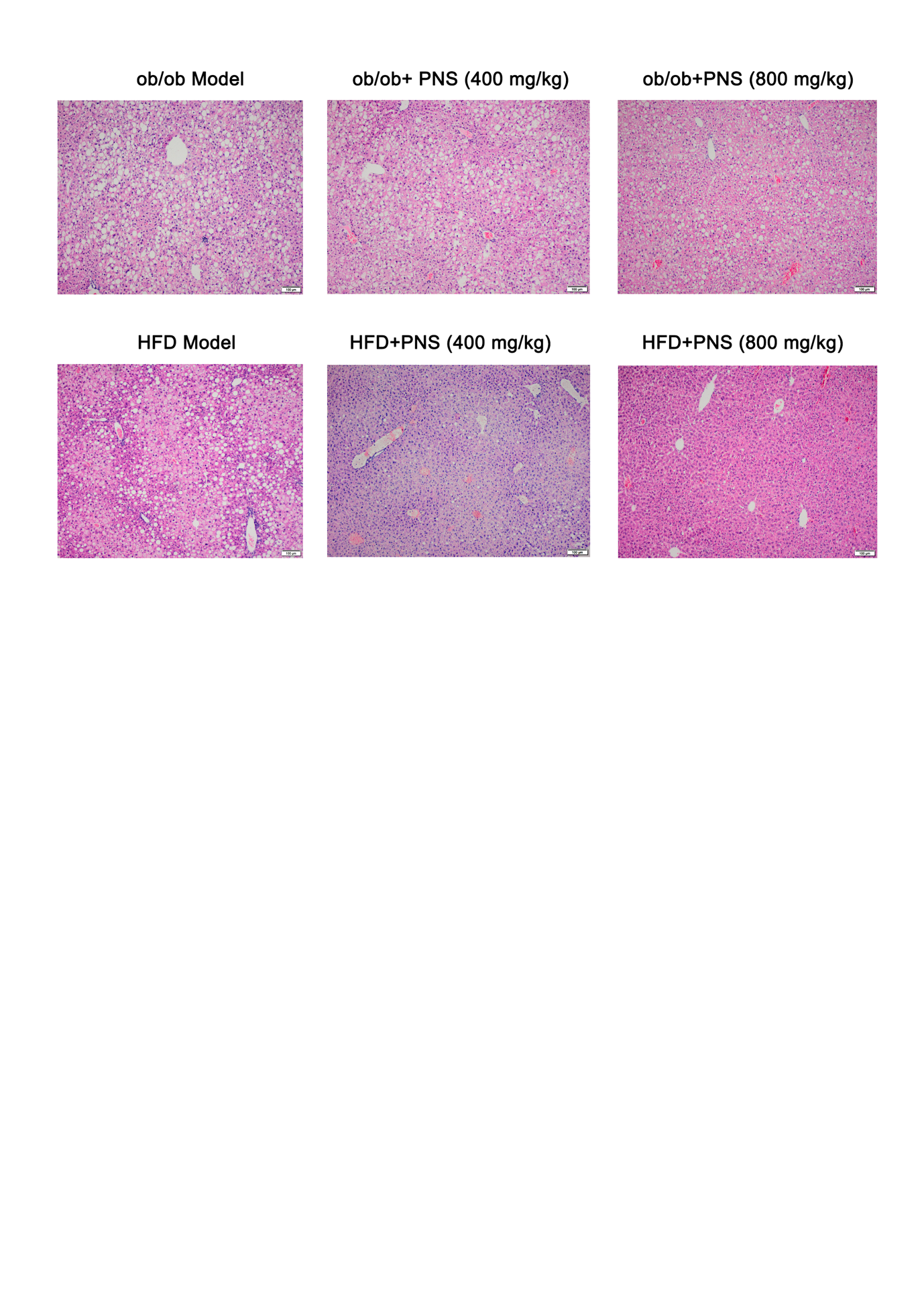


**Figure S2** The liver histology in ob/ob and HFD-fed mice treated with vehicle or PNS (400 mg/kg and 800 mg/kg)

Table S1. Real-time PCR primer sequence

| Gene | Sequence(5' to 3‘) | Sequence(5' to 3‘) |
| --- | --- | --- |
| ACC | GTCCCCAGGGATGAACCAATA | GCCATGCTCAACCAAAGTAGC |
| FAS | GCGATGAAGAGCATGGTTTAG | GGCTCAAGGGTTCCATGTT |
| SREBP-1C | GAACAGACACTGGCCGAGAT | GAGGCCAGAGAAGCAGAAGAG |
| PPARα | TCGGCGAACTATTCGGCTG | GCACTTGTGAAAACGGCAGT |
| PPARγ | GTACTGTCGGTTTCAGAAGTGCC | ATCTCCGCCAACAGCTTCTCCT |
| CPT-1 | CAGAGGATGGACACTGTAAAGG | CGGCACTTCTTGATCAAGCC |
| CD36 | GAACCACTGCTTTCAAAAACTGG | TGCTGTTCTTTGCCACGTCA |
| ACOX-1 | CGGAAGATACATCCCGGAGACC | AAGTAGGACACCATACCACCC |
| ACAT1 | GCAGGGAAGTTTGCCAGTGAGA | GAACACGGTCTTGAGCTTTGGC |
| ACAT2 | GAGATTGTGCCAGTGCTGGTGT | GTGACAGTTCCTGTCCCATCAG |
| ECH1 | CGTGACCTCATCAGCAAGTACC | GCAGTAGCGAATGTCACAGGCA |
| Collagen I | CGGGCAGGACTTGGGTA | CGGAATCTGAATGGTCTGACT |
| Collagen IV | CAACACTAACCACAGACTGAAT | GAAGGAGGCTAACAAAGGC |
| α-SMA | TGGCACCACACCTTCTACAA | CGGAGGCATAGAGGGACA |
| TNFα | CTACCTTGTTGCCTCCTCTTT | GAGCAGAGGTTCAGTGATGTAG |
| IL-6 | AGGATACCACTCCCAACAGACCT | CAAGTGCATCATCGTTGTTCATAC |
| CD14 | GGCCGCGCGGATTCCTAGTC | ATCGGGTCCGGTGGCTTCCA |
| β-actin | ACGGCCAGGTCATCACTATTG | TGGAAAAGAGCCTCAGGGC |
